# Does Maternal Depression Undermine Childhood Cognitive Development? Evidence from the Young Lives Survey in Peru

**DOI:** 10.3390/ijerph17197248

**Published:** 2020-10-03

**Authors:** Magdalena Bendini, Lelys Dinarte

**Affiliations:** The World Bank, Washington, DC 20433, USA; mbendini@worldbank.org

**Keywords:** child development, child vocabulary, maternal mental health, Peru

## Abstract

This paper studies the effect of maternal depression on early childhood cognition in Peru, where rates of depression are around 50%. By using an instrumental variables approach, this study exploits variation in the exogeneity of the exposure to shocks during early life to instrument for maternal depression. The empirical strategy exploits a novel longitudinal data—the Young Lives survey—that includes information on cognitive outcomes of children and variation in their mothers’ mental health status between rounds of data collection. Results suggest that maternal depression is detrimental to a child’s vocabulary at age 5, but effects fade out by age 8. Effects do not vary by maternal education but are significant only for children living in disadvantaged households. Estimations indicate that the presence of a partner worsens the effect of maternal depression on vocabulary development, results that are driven mainly by households with heavy-drinking partners. Our findings make a strong case for recognizing maternal mental health problems as disorders of public health significance and guide maternal and infant health policies in Peru.

## 1. Introduction

Maternal depression is a major global public health challenge due to its high prevalence and direct and indirect consequences. Globally, depression is experienced by about 10 percent of pregnant women, and by 13 percent of women who have just given birth [1]. In developing countries, the prevalence of depression is almost 50 percent higher than in developed contexts: Around 15.6 percent of women experience it during pregnancy and 19.8 percent after childbirth [1]. Given the limited availability of data on maternal depression in developing countries [2] and that it remains under-diagnosed and undertreated, these figures likely represent a lower bound of the scale of the problem.

Existing studies on maternal mental health warrant concern about the economic and human costs of maternal depression, not only to the women suffering from it but also to the children in their care, given the crucial role mothers traditionally play in childrearing, particularly when children are younger [3,4]. Maternal depression is characterized by sadness, negative affect, loss of interest in daily activities, fatigue, difficulty thinking clearly, and bouts of withdrawal and intrusiveness, and may interfere with the consistent, attentive, and responsive caregiving associated with effective parenting [5]. Because mother-child interactions during early life shape foundational neural circuits [6,7], neglect or maltreatment associated with maternal depression can undermine children’s brain development and lead to worse health (physical and mental), cognitive, and behavioral outcomes [8,9,10]. Given that it often “goes hand in hand with poverty” [6], a major concern about maternal depression is that it may increase poverty and contribute to its intergenerational transmission. In particular, maternal depression can intensify the negative effects of material deprivation and exposure to exogenous shocks associated with poverty, and confine children to substandard developmental trajectories and hence worse outcomes later in life. However, despite the potentially far-reaching harmful effects of maternal depression on mothers and child welfare, there is still a limited amount of rigorous evidence that quantifies its consequences on child development, the channels through which it acts, and how to mitigate its impact on children, particularly in developing countries.

The present paper aims to provide causal evidence of the effects of maternal mental health on children’s human capital accumulation in a developing country. We study the under-explored relationship between maternal depression and child cognition, a dimension of child development that has been extensively documented as a crucial determinant of life outcomes [11,12,13]. We focus our analysis on the context of Peru, a developing country with a high prevalence of maternal depression.

To shed light on the issue, we conducted our analysis using information from the Young Lives (YL) survey in Peru, a rich longitudinal household survey that follows households with at least one child born between 2001 and 2002 (index child). For our analysis, we used YL’s first three rounds: A baseline round in 2002, when the index child was 6–20 months old, the first follow-up when the child was 4–6 years old, and the last round in 2009–2010, when the child index was 7–8 years of age. The YL also has the novelty that includes questions related to maternal mental health and a child’s vocabulary, along with a wealth of information on child, family, and community characteristics.

Inspired by the literature that links the exposure to shocks during pregnancy, maternal mental health, and children’s outcomes, we employed an instrumental variable (IV) approach as an estimation strategy. This approach helps us to address the reverse causality bias in the estimation of the effect of maternal depression on a child’s vocabulary. We exploit the richness and longitudinal nature of the data to better capture the dynamic nature of maternal mental health on child cognitive development at the age of 5 and 8 years old. In particular, we instrument maternal depression with having experienced a shock (loss of crop or livestock) at baseline (when the child was in utero or recently born). We also strengthen the robustness of the analysis by considering variations of the indicator used for maternal depression, exploring heterogeneous effects of household characteristics, such as mother’s education, household wealth, and the presence of a male partner and some of his characteristics. Given the large set of potential controls, and to avoid overfitting the model or omitted variables bias, we used a machine learning procedure to select the instruments and controls to include in our model.

The remainder of the paper is organized as follows. In the next section, we describe our research design, including details on the data we are using for our analysis and some descriptive statistics. In Results, we present the main results, including some robustness checks. Finally, in Discussion and Conclusions, we discuss the policy implications, this paper’s contribution to the literature, and conclude.

## 2. Materials and Methods

In this section, we briefly described the data we used to assess the impact of maternal depression on early child vocabulary in Peru. Then, we presented some descriptive statistics of the different rounds of data used in our analysis. Since we restricted our study to the sample that had information across all rounds, we also briefly described the results from the sample attrition analysis.

### 2.1. Data

#### 2.1.1. Description

To measure the effects of MMH on child development, we used the first 3 rounds of the Young Lives Peru Survey (YL), conducted by the University of Oxford and core-funded by the UK Department for International Development. The YL survey was also being conducted in Vietnam, Ethiopia, and India (Andra Pradesh region). As of now, 5 rounds of data have been collected, which can be publicly accessible through the Young Lives website (https://www.younglives.org.uk/content/data-research). This was a rich longitudinal survey that included a complete set of individual, parental, household, and community characteristics, including early developmental, economic and demographic indicators, as well as information about social assistance programs in every community. The baseline sample of YL was cluster stratified, with 20 districts randomly selected across the country. Because the YL project was particularly interested in children living in poorer households, the sampling frame excluded the top 5 percent of districts as measured by a district poverty ranking. Despite excluding the least poor, it has been documented that the data reflects the Peruvian population in a broad range of indicators. Within each of the selected districts, 100 households with at least one child born between 2001 and 2002 (index child) were chosen randomly to participate in the project. Within each household, YL surveyed an index child who was born in 2000–2001 and was followed from infancy until they reached their mid-teens. The baseline round was conducted in 2002 when the index children were aged 6–20 months, the first follow-up conducted in 2006/2007, when they were between 4 and 6 years old, and the last round in 2009/2010, when they were between 7 and 8 years of age. The attrition rate between the 3 rounds of data collection was approximately 4 percent, which was low by international standards [14].

Of the 2000 index children in the baseline round, we focused our analysis on the sample of 1095 of them that were present in the first 3 waves for whom data on maternal mental health and Peabody Picture Vocabulary Test (PPVT) scores were available. We presented below tests for differences in some characteristics between the included and excluded samples.

#### 2.1.2. Measures of a Dimension of Child Development

We use PPVT scores [15] as the measure of early vocabulary skills, a strong predictor of later cognitive ability, including writing and reading skills, schooling, and labor market outcomes later in life [13,16,17,18]. In the YL survey, this outcome was measured using the Spanish version of the PPVT instrument. The PPVT measures receptive vocabulary; children are shown slides, each of which has 4 pictures, and were asked to identify the picture that corresponded to objects or actions named by the test administrator. Children did not need to name the objects or actions or be able to read or write them. It was just an object identification or association process. The test continued until the child had made 6 mistakes in the last 8 slides. The number and the level of difficulty of questions differed according to children’s age (see [19]). We, therefore, constructed age-specific z-scores by subtracting the month-of-age-specific mean of the raw score and dividing by the month-of-age-specific standard deviation. PPVT scores were available in the 2nd and 3rd rounds of the YL survey, i.e., when children were 4–6 and 7–8 years.

#### 2.1.3. Measures of Maternal Mental Health

The explanatory variable was constructed using the information on maternal common mental disorders from the Self Reporting Questionnaire 20 items (SRQ20), a screening (case-finding) tool included in the YL survey. The SRQ20 consisted of 20 yes/no questions with a reference period of the previous 30 days. The tool had a number of limitations, including the small number of items, the fact that it was not diagnostic, and could not separate out anxiety from depression. Still, the tool had been recommended by the World Health Organization and has acceptable levels of reliability and validity in developing countries. To the extent that depression and anxiety are closely related, and both of them can undermine the quality of care mothers provide to their children, the information gathered from the questionnaire was very valuable. Henceforth, we will use the term mental health to refer to both cases of depression and/or anxiety.

Using the responses to the questionnaire, we estimated 3 mental health indexes: The simple average of all items and 2 standardized items using factor analysis and principal components analysis. As we explain below, we used the information on maternal mental health from the first round of the YL survey.

#### 2.1.4. External Shocks

We exploited the availability of data on exposure to external shocks in the first round of the Peruvian YL. Caregivers were asked about events or changes that negatively affected the household welfare, and that occurred since the mother of the index child was pregnant until the day of the interview. The survey respondents described the event, and the enumerator classified it among the 14 categories. We grouped these categories into 6 groups of shocks, including natural disaster, crop or livestock loss, decrease in food availability, job or income loss, death or severe illness, and birth/new household member.

#### 2.1.5. Other Relevant Variables

In addition to the outcomes of interest and data on shocks, we used additional variables available in the survey that we used to address potential concerns to our identification strategy, as we explained in the following section. These additional variables consist of indexes that captured information on wealth, housing quality, and consumption of durable goods. These indexes were created using information reported by the caregivers. In each round, they were asked about the assets they own, characteristics of the household (materials of the floor, walls, etc.), among others. To collect consumption data, caregivers were asked how much they spent on non-food items during the last 30 days or on durable goods over the last 12 months.

### 2.2. Descriptive Statistics

Table 1 reports the summary statistics of the variables used in this paper for the sample under analysis. We separated the variables into 4 panels by mother, child, household, and community characteristics. Columns 1–3 presented mean, standard deviation, and the number of observations for the sample in the 2006/2007 round. Similarly, columns 4–6 showed the same statistics for the 3rd YL round (2009/2010).

As presented in Panel A, mothers were 31–35 years old on average between the 2 rounds. On average, 16% of these mothers reported being of indigenous origin, and although 79% of them reported they were literate, 57% had not completed primary school. Finally, statistics showed that 30% of mothers had mental health issues in 2002, and 94% of them reported that they attended antenatal care while they were pregnant from the index child. In terms of children’s characteristics (Panel B), half of index children were boys and 16% of them were the eldest. Cognitive outcomes, as measured by PPVT Z-scores, were practically unchanged between the 2 rounds, even if, as expected, the mean score increased as the children age, reflecting a larger vocabulary. The average child in the sample scored 0.06 standard deviations above the mean PPVT score of a reference child in both 2006/2007 and 2009/2010. Children’s height-for-age Z-scores, on the other hand, showed an improving trend.

To summarize information at the household level, we created some indexes that captured information on wealth, housing quality, and consumption of durable goods (see Panel C). Each of these indexes took values between 0 to 1. A household with an index level close to 0 (1) indicated that the family was worse (better) in the particular dimension that the index was measuring. In 2007, the average household in the sample under analysis was below the median of the distribution in all indexes. The wealth and housing quality indexes of the average household from our sample remained similar between the 2 rounds. Only the consumption of durable goods index increased between 2006–2009, which can be related to an increase in the number of older household members that consumed more expensive durable goods. Moreover, 58% of households under analysis lived in urban areas and had 5.5 members on average, 1.3 of them were school-aged children in 2006/2007. Three years after, 18% of households were more likely to live in urban areas.

Appendix A compared the sample under analysis with the observations excluded from the study. There are only 2 differences in maternal characteristics between these 2 sub-samples, and the difference remained statistically significant at 10%: Mothers in the sample were less likely to have completed primary school and were less likely to live in urban areas.

### 2.3. Empirical Strategy

What were the ways in which maternal depression can undermine children’s cognitive outcomes? We framed our analysis following Frank and Meara’s Model (FMM) [20] of maternal depression effects on the formation of children’s skill, which was inspired by Cunha and Heckman’s inter-generational model of human capability formation [13,21]. FMM assumed that a skill S was constituted in period *t*, through a production function *f* and several determinants that occurred in the previous period (*t* − 1). In sum, the model can be represented as follows:

(1) $$S_{t}=f\left(S_{t-1},I_{t-1},PS;M_{t-1},\right)$$

where S is the level of skill formation, PS represents parental skill attributes (education, cognitive abilities, etc.), $I_{t-1}$ indicates monetary and non-monetary investments in child capabilities, and $M_{t-1}$ is maternal mental health status at time *t* − 1. Mental health problems that interfered with mother-child interactions or undermined maternal behavior during *t* − 1 could potentially undercut the effectiveness of parental skills and/or reduce the productivity of investments and result in deficient children’s cognitive ability later in life.

To empirically estimate this theoretical model, we exploited information on maternal mental health during the 1st round and data on cognitive outcomes for our sample of 1095 children for which we have information of PPVT Z-scores from the 2nd and 3rd rounds of data collection. A naïve estimation of the effects of exposure to lagged maternal stress on cognitive development will regress a measure of maternal stress in 2002 on the PPVT Z-scores in 2006/2007 and 2009/2010, using the following specification:

(2) $$PPVT_{i,t}=\alpha_{0}+\alpha_{1}MH_{i,t-1}+\alpha_{2}C_{i,t}+\alpha_{3}M_{i,t}+\alpha_{4}H_{i,t}+\epsilon_{it}$$

where $PPVT_{i,t}$ represents the PPVT Z-scores for child *i* in period *t* (i.e., 2006/2007 or 2009/2010). $MH_{i,t-1}$ captures the value of any of the three maternal mental health indexes we estimated using data from 2002. $C_{i,t}$, $M_{i,t}$, and $H_{i,t}$ are vectors of child, mother, and household/community observable and time-varying characteristics that can lead to differences in cognitive ability across children and influence their parents’ investments in them. These vectors include all the variables presented in Table 1, all of which have been documented to affect children cognition (for a review, see [6]). $\epsilon_{it}$ represents a random, idiosyncratic error term.

Under the assumption of complete exogeneity of $MH_{i,t-1}$, the parameter of interest, ${\hat{\alpha }}_{1}$, measures performance in the PPVT at each period *t* for children whose mothers were depressed in 2002. The fact that the specification used measures of maternal depression and child’s vocabulary taken at different points in time addressed, to a large extent, the possibility of reverse causality. However, the probability that there were unobserved factors, such as pollution, access to services, or changes that had affected the household between rounds—that influenced maternal mental health and children’s outcomes cannot be entirely ruled out. Consequently, we used an instrumental variable (IV) approach to address the possibility of omitted variable bias.

In addition, the IV estimation helped to remedy the problem of measurement error in the main explanatory variable, which could be a relevant factor in the context of this paper. In particular, our main explanatory variable captured symptoms of mental health issues that affected mothers 30 days prior to the survey in 2002. We used those symptoms and estimated indexes of mental health, which constituted proxies of the unobserved, latent variable $MH_{i,t-1}^{*}.$ Thus, estimations of Equation (2) that incorporated the proxy for maternal depression can produce inconsistent estimators of $\alpha_{1}$ and lead to attenuation bias of these coefficients if $MH_{i,t-1}$ and the error term $\epsilon_{i,t}$ are negatively correlated [22,23].

The IV approach hinges on finding observable covariates that are correlated with maternal mental health, but which do not affect child cognitive status or other possible omitted variables. Considering this, we define our instrument by relying on the existing evidence that identifies the negative effect of exposure to exogenous shocks during pregnancy or during the first months after birth on children cognitive outcomes [3,24,25,26,27,28,29]. Some of these papers find that the main mechanism driving this relationship is maternal stress induced by the shock. Therefore, by exploiting the fact that the first round of YL asked caregivers about exposure to shocks, we use them to instrument maternal mental health. We excluded natural disasters and decreases in food availability due to lack of variation (less than 0.18% of households reported any of these shocks) and job or income loss because it can be highly correlated with the fact that the woman just gave birth. Hence, we restricted our analysis to the remaining three shocks–loss of crop or livestock, death or severe illness, or changes in their household composition—as potential instruments of maternal mental health. In this sense, Equation (2) corresponds to our second stage estimation, and our first stage will be given by the following:

(3) $$MH_{i,t-1}=\beta_{0}+\beta_{1}S_{i,t-1}^{j}+X_{i}+\epsilon_{i}$$

where $S_{i,t-1}^{j}$ indicates if the mother of child *i* was affected by shock *j* and $X_{i}$ represent the vectors of child, mother, and household characteristics described in Equation (2).

The validity of the instrument had to meet 2 conditions. First, it had to be relevant. In other words, the correlation between the shock and maternal mental health had to be high and statistically different from zero. To test this condition, we presented statistics of the shocks and measures of maternal mental health in Table 2, panels A and B. Panel C summarizes the correlations between each measure of maternal mental health and the three shocks under analysis. All correlations were statistically significant. In particular, the correlation between the loss of crop or livestock and the different indexes of maternal mental health ranges between 0.34 to 0.70.

The second condition for the instrument to be valid was exogeneity. In other words, suffering a shock during pregnancy or during the 1st months after birth should not have an impact on children’s vocabulary at the age of 5 other than through the impact on maternal mental health in the period when the shock occurred. There were 3 potential concerns that might affect this assumption, but we aimed to address those concerns with our specification. First, there was the concern of the nutritional effect of an income shock. A past shock can affect children’s nutritional status in *t* − 1, which can then translate into worse cognitive development later in life. To address this concern, we controlled for several children anthropometric measures. A 2nd concern was the learning resources: The shock could limit the exposure of the child to enriching opportunities or materials that might help her to improve her vocabulary development during childhood. To control for this potential channel, we included in our specification some measures of household wealth and consumption in *t* − 1. Finally, the 3rd concern was that the shock limited additional stimulation that might have been provided to her by other members in the household, in addition to the mother and her partner. For example, in extended households, non-working relatives tended to contribute to childcare duties. The shock may forced these other household members to find a job, which could, in turn, limit opportunities for child stimulation and consequent development. Since extended households were larger than the non-extended ones, we controlled for that characteristic by including the variable household size in our model. Alternatively, we tested the exogeneity assumption in our model by estimating the correlation between the measure of vocabulary and the shock, conditional on the variables that captured differences in availability of learning resources, child’s nutritional status, and the rest of the control variables. These results are presented in Appendix B.

Finally, having at least three instruments and a large set of potential control variables posited the challenge of selecting the “right” set of them. On the one hand, using too few controls or the wrong ones may lead to omitted variable bias. However, by using too many, our model may be affected by overfitting. To address this issue, we estimate the parameters of interest using the Instrumental Variables Least Absolute Shrinkage and Selection Operator (IV-LASSO), a routine for estimating structural parameters in linear models with many controls and/or instruments. In particular, we used the post-double selection (PDS) methodology [30,31] that was applied in Stata’s built-in commands by Ahrens et al. [32].

## 3. Results

We found three main results. First, maternal depression was detrimental to a child’s vocabulary at the age of 5, but the effect faded out by age 8. Our estimations indicated that 1 standard deviation of maternal depression during pregnancy and postpartum reduced the vocabulary of 5-year-old children—measured through PPTV scores—in 0.54 standard deviations. This impact is no longer statistically significant at the age of 8, even considering different measures of maternal depression. The magnitude of these effects is large and consistent with the upper bound found in the existing literature.

Second, heterogeneity analysis by household wealth shows that these effects were driven by children living in disadvantaged households. When the impact of maternal depression was analyzed separately by household wealth, there was evidence of worse effects for less wealthy households, providing suggestive evidence that maternal mental illness may contribute to the intergenerational transmission of poverty given the high rates of depression among low-income mothers cited in the literature.

Finally, we explored if the presence of household members that supported mothers can dampen the effect of experiencing a shock that affects maternal mental health. We focused on the presence of a partner in the household when the woman was pregnant or during the first year after childbirth. Our estimations indicated that mental health issues of women living with a partner when they experienced a shock effect more their child’s vocabulary than those without a partner. Upon further exploration, we found that women living with heavy-drinking partners were the ones driving the negative impacts on the child’s cognitive development. In this sense, this set of results indicates that it is not the presence of a partner in itself that matters, but the quality of such partners.

### 3.1. Main Results

Table 3 reports the main results of the paper. Columns 1–3 present results for Ordinary Least Squares (OLS) with PDS-selected variables and full regressor set. Each column shows the results for a measure of mental health, as defined above. Columns 4–6 show results for the IV with PDS-selected variables and full regressor set as depicted in Equation (2).

First-stage estimates for the exposure to an external shock on maternal mental health are presented in Panel B. Selected instrument by the LASSO regression was suffering crop or livestock loss during pregnancy or within the first year of the index child. The outcome variable was a measure of maternal mental health in 2002. The coefficient indicated changes in maternal mental health after experiencing a shock of crop or livestock loss during pregnancy or after giving birth. Across columns, the precision of the estimate does not change, but the size of the coefficients is sensible to the measure of mental health used.

As presented in Panel A, our IV estimations indicate that poor maternal mental health has a negative impact on child cognition. An increase by one standard deviation in maternal mental health problems when children were 1-year-old or younger was associated with a reduction of 0.5–0.54 standard deviations in vocabulary Z-scores when children were 5 years old. This effect corresponds to a reduction of 31 percent of the mean PPVT raw score. These large estimated effects were consistent with existing evidence. For example, Aizer et al. [3] found that exposure to stress hormones in utero negatively affects cognition (verbal IQ at age 7), behavior, and motor development. Specifically, the authors found that exposure to cortisol in the top quintile of the distribution was associated with a 43 percent of a standard deviation reduction in verbal IQ.

The LASSO regression selected the following controls: Mother’s age, wealth index, living in an urban area, child’s age, consumption of durable goods index, household size, number of children younger than 5 years in the household, and height for age Z-score. The effects on child cognition of demographic controls (not shown in the table) are in the expected direction. Z-scores of children living in urban areas were higher than those of children living in an urban area. In addition, children’s nutritional status also affected performance in the PPVT. The coefficients for wealth were positive, statistically significant, and among the highest, which was in line with research that points to socioeconomic status gradients of cognition as measured by vocabulary [16,33,34,35].

We then explored if these negative impacts were held three years after the first measure of vocabulary. Using data of child vocabulary at the age of 8, we estimated the model presented in Equations (2) and (3). The main results are presented in Table 4. Our estimations showed that maternal depression had no effect on the child’s vocabulary at the age of 8. Not only were the estimated coefficients not statistically significant, but also their sizes were very small—that is, the vocabulary of children whose mothers’ experienced mental health problems when they were 1-year-old caught up with the vocabulary of children whose mothers did not suffer mental health problems. As we present in the table in Appendix D, the effects of a shock on maternal mental health are not statistically significant a year after the woman experienced the shock. These results suggest that the effect of exposure to maternal depression during early childhood need not undermine language development permanently, and exposure to rich vocabulary environments later on during childhood can compensate for earlier developmental gaps. For our sample, it is possible that the convergence in vocabulary development is explained by the fact that by the time they reached age 8, all children had had exposure to formal education opportunities (99.9% of children in our sample), which may have a compensatory effect on children’s vocabulary development. Still, given that early vocabulary constitutes a foundational skill that facilitates the development of other cognitive skills, based on our results, we cannot rule out the possibility that exposure to maternal depression during early life does not undermine cognitive development and academic achievement.

### 3.2. Heterogeneity by Household Characteristics

We explore heterogeneous effects by a number of maternal characteristics that have been identified in the literature as moderators of the effect of mental health, using our main model and all measures of mental health.

First, we run separated regressions by different levels of household wealth, which, it is generally agreed, influences the extent to which maternal mental health affects children [3,6,36]. For our analysis, we compared the vocabulary of children living with mothers with different mental health levels within the upper or lower half of the wealth distribution. Using data from the 2002 round, we estimated three indexes: Housing quality, consumer durables, and services indexes. Then, we created a wealth index for each household of our sample that consisted of the average of the three first ones mentioned above. Using the wealth index distribution, we separated our sample by the median of the wealth index distribution. The results are presented in Table 5. Our estimations indicate that 1 standard deviation of maternal mental health issues reduces vocabulary by 0.58 to 0.63 standard deviations of children living in less wealthy households (columns 4–6). This is around 0.08 standard deviations more than the impacts in the total sample. The effects of maternal mental health on the vocabulary of children living in wealthier households are not statistically significant.

These results are an important contribution to the evidence of intergenerational transmission of poverty. Poor households are less able to protect themselves from external shocks, such as crop or livestock losses, which then increases stress levels for household heads. In low-income families where there is a pregnant woman or with a child younger than 1-year-old, our results indicate that the negative shock translates into a reduction in the child’s cognitive skills in the short term. Giving that the development of these skills during early childhood is the foundation of future ones [13,16,17,18], this negative effect can have long-lasting impacts in terms of human capital accumulation, which is in line with existing literature indicating that events before five years old can have large long-term impacts on adult outcomes [3,37].

The existing literature has also found that maternal schooling levels may modulate the impact of depression [6,38]. A recent paper by Aizer et al. [3] finds that mothers with low levels of human capital are characterized by higher stress levels and that the negative impact of their elevated stress levels on their children is greater. We explore this heterogeneity using the YL data by separating the sample into two groups: Mothers with less than primary education and mothers with at least primary education completed. Our estimations are presented in Table 6. Unlike the existing literature, there are no apparent differences in the effect of maternal mental health for mothers who have completed or not primary education.

However, when we combine the heterogeneity by household wealth and maternal education, we find out that our results are still in line with the existing literature for two reasons. First, the papers finding that lower maternal education exacerbates the negative effect of maternal mental health on early vocabulary argue that this low maternal education can be associated with sub-optimal childcare practices or to restricted access to quality material inputs and opportunities. Access to quality inputs that help to improve children’s vocabulary is restricted to less wealthy households as well. Second, there is extensive evidence of a strong correlation between a mother’s education and socioeconomic status. In this sense, the expected differences in terms of lack of resources that allow overcoming the negative effects of maternal mental health on child language are captured not by maternal education but by household wealth in this particular context.

In addition, we explore whether the effects of exposure to maternal mental health issues during a child’s young age varies depending on whether the mother has a partner, given that this factor may modulate the impact of depression. The literature suggests that the presence of other members in the household that provide support to the mother can buffer the effect of depression on children. Our results are presented in Table 7. Our estimates suggest that having a partner can actually worsen the negative effects of maternal mental health issues on a child’s vocabulary (Columns 1–3).

To understand these unexpected results, we further explore the characteristics of the partner. First, we separate the sample of women living with a drinking (columns 4–6) and a heavily-drinking partner (columns 7–9). Our estimations indicate that Z-scores of children with mothers whose mental health was 1sd worse at *t* − 1 and lived with a drinking partner was, on average, 0.52 to 0.56 standard deviations lower than Z-scores of children of mothers who were also living with a drinking partner. These coefficients were statistically significant at the five percent level. Moreover, the estimated coefficients for the effects of mentally ill mothers living with heavily-drinking partners indicated that this group was driving the effects described before.

A potential explanation of these results was the alcohol-induced physical intimate partner violence (AIPIPV). Existing evidence from psychology shows that Intimate Partner Violence (IPV) is a major predictor of post-traumatic stress disorder in abused women [39] and can drive to negative interactions between mother and children [40], directly affecting child development [41]. Using YL data, Bedoya et al. [41] found that IPV was one of the main forms of violence against women in Peru. The authors also found that early-life exposure to AIPIPV was indeed associated with lower test scores in vocabulary.

A second explanation was budget constraints. Allocating household income to consume alcohol reduces the availability of resources in the household for other needs, including inputs that help to support child development. This creates a vicious cycle for mothers with mental health issues since it can impose additional stressors.

## 4. Discussion

Results in this paper underscore children’s incredible resilience, while at the same time provide further evidence that maternal depression can undermine children’s development. Moreover, the heterogeneous findings by household wealth and quality of partner, combined with extensive evidence in the literature of the disproportionally high prevalence rates of anxiety and depression among households with low socio-economic status around the world cited in this paper’s introduction, suggests that maternal mental illness may contribute to the intergenerational transmission of poverty. In addition, stress, in general, and associated maternal mental illness in particular, constitute yet another pathway from poverty to substandard developmental trajectories and potentially worse outcomes later in life.

What are the implications of these findings for policymakers? To the extent that the maternal depression-child cognitive development relationship is causal, findings suggest that a two-pronged approach may be necessary for protecting children’s cognitive development from maternal depression. First, given its disease burden and the associated deleterious effects, a strong case can be made for recognizing maternal mental health problems as disorders of public health significance and integrated as such into maternal and infant health policies [38]. For this to occur, the public health commitment to mental health problems should increase, particularly in developing countries, where the current commitment is minimal [42].

Cost-effective interventions to effectively treat mental health issues that affect women in poorer households have been successfully implemented in developed and developing countries. Most relevant to this paper, evaluations of interventions that, in addition, to addressing maternal depression, also included children reported improved mother-infant interaction and better cognitive development [43]. Considering cultural differences and local sensitivities, similar initiatives could prove effective and efficient in improving maternal mental health in developing countries such as Peru and improving the livelihoods of children whose early development is hindered by maternal depression.

The heterogeneous results in this paper suggest that the child cognition nexus is a complex one, determined not only by maternal illness but also maternal and household characteristics that interact in ways that are not yet fully understood. Consequently, the most effective way to protect children’s welfare may be to target children themselves and build support systems at the household, community, or institutional level that protect vulnerable children’s outcomes. Programs and policies that promote poor children’s cognitive development directly, such as by improving access to quality pre-school programs or indirectly, by promoting cognitive stimulation at home and improving the quality of their home environments, may help prevent and compensate for early deficits related to maternal depression. In addition, given the hierarchical and interdependent nature of development, the earlier in life the intervention, the better. In recent years, there have been a number of interventions in Latin America that have successfully boosted the cognitive development of poor young children, including cash transfers to very poor households in Nicaragua [44], programs that increase preschool availability in Argentina and Uruguay [45,46], and a program of home visits in Colombia [47].

### Contributions to the Global Literature on Early Child Development

This paper makes several contributions to the literature that studies how parents influence children’s developmental outcomes. First, it uniquely identifies the impact of maternal depression on child cognition in a developing country, which, to our knowledge, has not been done before, as previous studies have focused on the effects of maternal depression on child health outcomes [48]. Findings from previous research of the effect of maternal depression on cognitive development are mixed and mostly use data from developed countries. In a study in England [14], the authors found that children of mothers who were depressed in the first year had reliably lower cognitive skills as measured by a test score at age 4 than children whose mothers had not been ill. Petterson and Albers [15] also reported lower cognitive outcomes for children exposed to depression in the U.S. In addition, Kurstjens and Wolke [16] concluded that maternal depression is linked with a higher probability of long-term effects for boys and neonatal risk born, chronic cases of depression, or if the family is exposed to other social risks. Our study, which focuses on Peru, provides much needed empirical evidence of the deleterious impact of maternal depression on child cognition in a region where the causal nexus between maternal depression and child cognition has not been studied before despite maternal depression prevalence rates that range between 35% and 50% [17].

Second, the paper focuses on an important marker of early cognition, the accumulation of vocabulary, which has been extensively shown to predict reading comprehension throughout school and into early adulthood [18]. To capture vocabulary competence, we use performance in the Peabody Picture Vocabulary Test (PPVT), a test of receptive vocabulary, which has been widely used and translated to Spanish and Quechua, the two most widely spoken languages in Peru.

Finally, this paper contributes to the literature on the protective effect that other household members can have on the development of children exposed to maternal depression. Our results suggest that, in and of itself, the presence of other household members does not attenuate the effect of maternal depression on child vocabulary development. In fact, the presence of heavy-drinking partners appears to worsen the effect of maternal depression. This latter result may be explained by the increased risk of Intimate Partner Violence (IPV) associated with high alcohol consumption. Evidence from psychology suggests that IPV constitutes a major predictor of post-traumatic stress disorder in abused women [39] and can lead to negative interactions between mother and children [40], directly affecting child development [41]. Using YL data, Bedoya et al. [41] found that IPV was one of the main forms of violence against women in Peru. The authors also found that early-life exposure to alcohol-induced IPV was indeed associated with lower test scores in vocabulary. Our results suggest that maternal depression is a mechanism through which alcohol-induced IPV leads to worse child vocabulary outcomes.

## 5. Conclusions

In this paper, we explore the extent to which maternal depression affects child cognition in Peru. The identification strategy exploits variation in the exogeneity of the exposure to a particular shock between pregnancy and when the child was 1 year old. Exposure to shock can affect maternal mental health and children’s vocabulary development. The paper’s main results indicate that exposure to a crop or livestock loss in 2002 increases maternal depression in that period. Moreover, a standard deviation of maternal mental health in 2002 negatively affects a child’s vocabulary up to 0.54 standard deviations when children are 5 years old, a result that fades out by the children are 8 years old. That is, our results suggest the negative effects of maternal depression on child receptive vocabulary do not persist beyond children’s early school years. However, given that vocabulary size in kindergarten and earlier predicts reading comprehension throughout school and into early adulthood, facilitating the development of other cognitive skills [18], we cannot rule out the possibility that exposure to maternal depression during early life does not undermine other markers of cognitive development in the medium to long term.

In addition to the main results discussed above, this paper also estimates heterogeneous effects by household wealth, maternal education level, and the presence of a partner in the household. When the impact of maternal depression is analyzed separately by household wealth, we find that the effects of maternal mental health issues are worse for children living in less wealthy households during the period when the shock occurred. These results shed light on the negative complementarities between poverty and maternal mental health. Somewhat surprisingly, we found no heterogenous effects by maternal education. Given that our estimations control for a host of important household characteristics that tend to be associated with maternal education (household wealth, consumption, size, number of young children), our results suggest that maternal education may not be the main conduit through which maternal depression undermines children’s vocabulary development.

The heterogeneity analysis, in terms of whether the mother has a partner, is enlightening. We find that having a partner does not attenuate the effect of maternal depression on child vocabulary, a result that is driven mostly by partners that are heavy alcohol drinkers. This result is consistent with the literature on domestic violence, which defines low-quality partners as those reported to consume high quantities of alcohol. This literature argues that having a drinking partner is positively correlated with IPV, maternal stress, and worse child vocabulary outcomes. Our results suggest that maternal depression is a mechanism through which alcohol-induced IPV leads to worse child vocabulary outcomes.

## Figures and Tables

**Table 1:** Descriptive Statistics by Survey Round.

Variables	(1)	(2)	(3)	(4)	(5)	(6)
	Year 2006/2007			Year 2009/2010		
	Mean	S.D.	N	Mean	S.D.	N
Panel A. Maternal characteristics						
Age of the mother (years)	31.43	6.64	1095	33.71	6.64	1095
Indigenous ethnic group	0.16	0.37	1095	−	−	−
Less than primary school	0.57	0.50	1095	−	−	−
Literate	0.79	0.41	1095	−	−	−
Attended antenatal care in 2002	0.94	0.23	1095	−	−	−
Mother has Mental Health Problems in 2002	0.30	0.46	1095			
Panel B. Child characteristics						
Child is a boy	0.50	0.50	1095	−	−	−
Weight at birth (kg)	3.21	0.51	1095	−	−	−
Long-term health problems	0.09	0.09	1095	−	−	−
Age (in months)	63.5	4.71	1095	94.9	3.58	1095
Child is the eldest	0.16	0.37	1095	0.23	0.42	1095
Height for age Z-score	−1.42	1.08	1095	−1.05	1.02	1095
PPVT score (raw)	29.9	17.4	1095	47.6	12.9	1095
PPVT Z-score	0.06	0.98	1095	0.07	0.95	1095
Panel C. Household characteristics						
Wealth index	0.49	0.22	1095	0.56	0.20	1095
Housing quality index	0.41	0.24	1095	0.44	0.24	1095
Consumption of durable goods index	0.37	0.23	1095	0.45	0.23	1095
Live in urban area	0.58	0.49	1095	0.76	0.43	1095
Household size	5.52	2.13	1095	5.44	1.94	1095
School aged children in the household (*n*)	1.33	1.25	1095	1.09	1.05	1095
Panel D. Community characteristics						
Violent crime in community	0.33	0.47	1095	0.36	0.48	1095
Social assistance (education) available	0.95	0.22	1095	0.98	0.13	1095

Table 1 present summary statistics (mean and standard deviation) of the variables used in the analysis. These variables are available in the first three rounds of the Peruvian Young Lives Survey. The sample is restricted to children with available information on maternal mental health in 2002 and PPVT scores in 2006 and 2009.

**Table 2:** Correlations between Shocks and Maternal Mental Health Indexes (MHI) in 2002.

	(1)	(2)	(3)
Panel A. Descriptive statistics of Maternal MHI	Mean	S.D.	N
MHI-1	−0.02	0.50	1095
MHI-2	−0.04	0.98	1095
MHI-3	−0.04	0.92	1095
Panel B. Shocks experienced by mothers during pregnancy or within the first year after the child was born (in 2002)	Mean	S.D.	N
Crop or livestock loss	0.03	0.16	1095
Death, severe illness, divorce	0.13	0.34	1095
Birth/new household member	0.06	0.24	1095
Panel C. Correlations between shocks and Maternal MHI in 2002	MHI-1	MHI-2	MHI-3
Crop or livestock loss	0.34 ***	0.70 ***	0.65 ***
Death, severe illness, divorce	0.15 ***	0.32 ***	0.29 ***
Birth/new household member	0.13 **	0.27 **	0.24 **

Table 2 presents summary statistics (mean and standard deviation) of maternal mental health indexes and shocks experienced by mothers of our sample of analysis. These variables are available in the first round of the Peruvian Young Lives Survey (2002). The sample is restricted to children with available information on maternal mental health in 2002 and PPVT scores in 2006 and 2009. Mental health index 1 is the standardized average of the SRQ-20 items. Panel A presents statistics of mental health indexes. Mental health index 2 and 3 are standardized indexes estimated using principal components and factor analysis, respectively. Panel B presents the % of mothers reporting being exposed to any of the four shocks. Panel C shows correlations between the mental health indexes and shocks. *** and ** indicate statistical significance at 1% and 5%, respectively.

**Table 3:** Effect of maternal mental health on children’s vocabulary at age 5.

Dependent Variable: Standardized PPVT						
	(1)	(2)	(3)	(4)	(5)	(6)
Panel A. Estimated coefficients from OLS and IV estimation approaches	OLS with PDS-selected variables and full regressor set			IV with PDS-selected variables and full regressor set		
	MHI-1	MHI-2	MHI-3	MHI-1	MHI-2	MHI-3
Maternal Mental Health	−0.0314	−0.0157	−0.0173	−1.025 *	−0.499 *	−0.536 **
	(0.0513)	(0.0254)	(0.0275)	(0.527)	(0.255)	(0.274)
Observations	1095	1095	1095	1095	1095	1095
Mother controls	Yes	Yes	Yes	Yes	Yes	Yes
Child controls	Yes	Yes	Yes	Yes	Yes	Yes
Household controls	Yes	Yes	Yes	Yes	Yes	Yes
Panel B. First-stage estimation				MHI-1	MHI-2	MHI-3
Shock: Crop or livestock loss				0.573 ***	0.616 ***	0.300 ***
				(0.165)	(0.179)	(0.088)
Observations				1095	1095	1095
Mother controls				Yes	Yes	Yes
Child controls				Yes	Yes	Yes
Household controls				Yes	Yes	Yes
Weak identification F-Stats (Full IV set)				11.53	11.69	11.75

Panel A in Table 3 presents the estimated impacts of maternal mental health on children vocabulary at the age of 5 years obtained from Equation (2), using Ordinary Least Squares (OLS) and Instrumental Variables (IV) as estimation approaches. The dependent variable was measured using the standardized value of the PPVT test. Mental health indexes are standardized values of the SRQ-20 items using three different estimation approaches. Columns (1–3) present estimated coefficients using OLS and columns (4–6) show coefficients using IV. Both approaches were implemented using the option PDS-selected variables and full regressor available in the LASSO command. The selected instrument was suffering crop or livestock loss during pregnancy or within the first year of the index child. Selected controls are mother’s age, wealth index, living in an urban area, child’s age, consumption of durable goods index, household size, number of children younger than 5 years in the household, and height for age Z-score. The sample is restricted to children with available information on maternal mental health in 2002 and PPVT scores in 2006 and 2009. *, ** and *** indicate statistical significance at 10%, 5% and 1%, respectively. Confidence intervals at the 95% confidence are presented in Appendix C. Robust standard errors in parentheses.

**Table 4:** Effect of maternal mental health on children’s vocabulary at age 8.

Dependent Variable: Standardized PPVT						
	(1)	(2)	(3)	(4)	(5)	(6)
Panel A. OLS and IV second-stage estimations	OLS with PDS-selected variables and full regressor set			IV with PDS-selected variables and full regressor set		
	MHI-1	MHI-2	MHI-3	MHI-1	MHI-2	MHI-3
Maternal Mental Health	0.006	0.006	0.008	−0.065	−0.032	−0.034
	(0.0471)	−0.0233	(0.0253)	(0.501)	(0.245)	(0.265)
Observations	1095	1095	1095	1095	1095	1095
Mother controls	Yes	Yes	Yes	Yes	Yes	Yes
Child controls	Yes	Yes	Yes	Yes	Yes	Yes
Household controls	Yes	Yes	Yes	Yes	Yes	Yes
Panel B. First-stage estimation				MHI-1	MHI-2	MHI-3
Shock: Crop or livestock loss				0.338 ***	0.689 ***	0.639 ***
				(0.091)	(0.184)	(0.172)
Observations				1095	1095	1095
Mother controls				Yes	Yes	Yes
Child controls				Yes	Yes	Yes
Household controls				Yes	Yes	Yes
Weak identification F-Stats (Full IV set)				13.61	13.65	11.75

Table 4 presents the effects of maternal mental health on children’s vocabulary at the age of 8 years. The dependent variable was measured using the standardized value of the PPVT test. Mental health indexes are standardized values of the SRQ-20 items using three different estimation approaches. Columns (1–3) present estimated coefficients using OLS and columns (4–6) show coefficients using IV. Both approaches were implemented using the option PDS-selected variables and full regressor available in the LASSO command. The selected instrument was suffering crop or livestock loss during pregnancy or within the first year of the index child. Selected controls are mother’s age, the mother is indigenous, mother literacy, wealth index, living in an urban area, consumption of durable goods index, and height for age Z-score. *, ** and *** indicate statistical significance at 10%, 5% and 1%, respectively.

**Table 5:** Heterogeneous effects of Maternal Mental Health on Children Vocabulary at Age 5 by HH Wealth Level.

Dependent Variable: Standardized PPVT						
	(1)	(2)	(3)	(4)	(5)	(6)
	IV with PDS-selected variables and full regressor set					
	Effects on children from wealthier HH			Effects on children from less wealthy HH		
	MHI-1	MHI-2	MHI-3	MHI-1	MHI-2	MHI-3
Maternal Mental Health	−2.971	−1.427	−1.509	−1.194 *	−0.581 *	−0.625 *
	(4.212)	(2.009)	(2.111)	(0.661)	(0.319)	(0.342)
Constant	1.098	1.101	1.098	1.147 **	1.157 **	1.158 **
	(0.869)	(0.854)	(0.839)	(0.583)	(0.576)	(0.574)
Observations	514	514	514	581	581	581
Mother controls	Yes	Yes	Yes	Yes	Yes	Yes
Child controls	Yes	Yes	Yes	Yes	Yes	Yes
Household controls	Yes	Yes	Yes	Yes	Yes	Yes

Table 5 presents estimated effects of maternal mental health on child vocabulary at age 5, separated by whether the HH is in the upper or lower wealth half of the distribution. We estimate the model selected from LASSO procedure. The selected control regressor set includes the mother’s age, wealth index, living in an urban area, child’s age, consumption of durable goods index, household size, number of children younger than 5 years in the household, and height for age Z-score. The sample is restricted to children with available information on maternal mental health in 2002 and PPVT scores in 2006 and 2009. Robust standard errors in parentheses. * and ** indicate statistical significance at 10%, and 5%, respectively.

**Table 6:** Heterogeneous effects of maternal mental health on children’s vocabulary at age 5 by the educational level of the mother.

Dependent Variable: Standardized PPVT						
	(1)	(2)	(3)	(4)	(5)	(6)
	IV with PDS-selected variables and full regressor set					
	Effects on children from mothers with primary incomplete			Effects on children from mothers with at least primary education complete		
	MHI-1	MHI-2	MHI-3	MHI-1	MHI-2	MHI-3
Maternal Mental Health	−0.799	−0.389	−0.419	−1.829	−0.889	−0.949
	(0.513)	(0.249)	(0.268)	(1.848)	(0.885)	(0.937)
Constant	1.292 ***	1.299 ***	1.300 ***	0.739	0.736	0.739
	(0.401)	(0.398)	(0.397)	(0.778)	(0.771)	(0.763)
Observations	719	719	719	376	376	376
Mother controls	Yes	Yes	Yes	Yes	Yes	Yes
Child controls	Yes	Yes	Ye	Yes	Yes	Yes
Household controls	Yes	Yes	Yes	Yes	Yes	Yes

Table 6 presents the estimated effects of maternal mental health on child vocabulary at age 5, separated by whether the mother has completed at least primary education or not. We estimate the model selected from LASSO procedure. The selected control regressor set includes the mother’s age, wealth index, living in an urban area, child’s age, consumption of durable goods index, household size, number of children younger than 5 years in the household, and height for age Z-score. The sample is restricted to children with available information on maternal mental health in 2002 and PPVT scores in 2006 and 2009. Robust standard errors in parentheses. *** indicates statistical significance at 1%.

**Table 7:** Heterogeneous effects of maternal mental health on children vocabulary at age 5 by the mother’s marital status and partner’s drinking behavior.

Dependent Variable: Standardized PPVT									
	(1)	(2)	(3)	(4)	(5)	(6)	(7)	(8)	(9)
	IV with PDS-selected variables and full regressor set								
	Effects on children from mothers with a partner			Effects on children from mothers with a drinking partner			Effects on children from mothers with a heavily-drinking partner		
	MHI-1	MHI-2	MHI-3	MHI-1	MHI-2	MHI-3	MHI-1	MHI-2	MHI-3
Maternal Mental Health	−0.889 *	−0.433 *	−0.465 *	−1.070 **	−0.518 **	−0.556 **	−1.150 **	−0.549 **	−0.587 **
	(0.459)	(0.222)	(0.239)	(0.511)	(0.246)	(0.264)	(0.573)	(0.270)	(0.287)
Constant	1.144 ***	1.147 ***	1.148 ***	1.195 ***	1.201 ***	1.200 ***	1.653 ***	1.642 ***	1.633 ***
	(0.339)	(0.337)	(0.336)	(0.379)	(0.376)	(0.375)	(0.439)	(0.434)	(0.432)
Observations	963	963	963	770	770	770	486	486	486
Mother controls	Yes	Yes	Yes	Yes	Yes	Yes	Yes	Yes	Yes
Child controls	Yes	Yes	Yes	Yes	Yes	Yes	Yes	Yes	Yes
Household controls	Yes	Yes	Yes	Yes	Yes	Yes	Yes	Yes	Yes

Table 7 presents the estimated effects of maternal mental health on child vocabulary at age 5, separated by whether the mother lives with a partner and his drinking likelihood. We estimate the model selected from LASSO procedure. The selected control regressor set includes the mother’s age, wealth index, living in an urban area, child’s age, consumption of durable goods index, household size, number of children younger than 5 years in the household, and height for age Z-score. The sample is restricted to children with available information on maternal mental health in 2002 and PPVT scores in 2006 and 2009. Robust standard errors in parentheses. *, ** and *** indicate statistical significance at 10%, 5% and 1%, respectively.

## Appendix A

**Table A1:** Tests for differences between included and excluded subsamples.

	(1)	(2)	(3)
Variables	Mean and Tests for Differences in Means between Included and Excluded Samples		
	Included	Excluded	*p*-Value
Panel A. Maternal characteristics			
Age of the mother (years)	31.43	33.73	0.235
Indigenous ethnic group	0.16	0.16	0.995
Less than primary school	0.57	0.42	0.085
Literate	0.79	0.77	0.567
Attended antenatal care in 2002	0.94	0.92	0.734
Panel B Child characteristics			
Child is a boy	0.50	0.51	0.847
Weight at birth	3.21	3.20	0.835
Long-term health problems	0.09	0.08	0.123
Age (in months)	63.5	63.42	0.568
Child is the eldest	0.16	0.17	0.723
Height for age Z-score	−1.42	−1.62	0.167
Panel C Household characteristics			
Wealth index	0.49	0.49	0.934
Housing quality index	0.41	0.42	0.76
Consumption of durable goods index	0.37	0.35	0.582
Live in urban area	0.58	0.62	0.073
Household size	5.52	5.60	0.382
School-aged children in the household (*n*)	1.33	1.32	0.923
Panel D. Community characteristics			
Violent crime in community	0.33	0.35	0.634
Social assistance (education) available	0.95	0.97	0.913

Table A1 presents the mean of the variables used in the analysis from the included sample (children with available information on maternal mental health in 2002 and PPVT scores in 2006 and 2009) and excluded one (the rest of the sample).

## Appendix B

**Table A2:** Test for exogeneity assumption of the IV.

Dependent Variable: Standardized PPVT	
Shock: Crop or livelihoods loss	
Coefficient	−0.194
Standard error	(0.117)
CI: Upper limit	0.036
CI: Lower limit	−0.424
Observations	1095
R-squared	0.362

Table A2 presents an alternative test for exogeneity of the instrument. We estimate a model that tests the independence between the shock under analysis (instrument) and the vocabulary measure, conditional on all variables that account for the availability of learning resources and child’s nutritional status. The estimated coefficient is not statistically significant. CI stands for “confidence interval.” R-squared indicates that 36% of the variance for the vocabulary measure is explained by the shock and the rest of independent variables.

## Appendix C

**Table A3:** Confidence intervals of Table 3.

Dependent Variable: Standardized PPVT						
	(1)	(2)	(3)	(4)	(5)	(6)
Panel A. Estimated coefficients from OLS and IV estimation approaches	OLS with PDS-selected variables and full regressor set			IV with PDS-selected variables and full regressor set		
	MHI-1	MHI-2	MHI-3	MHI-1	MHI-2	MHI-3
Upper bound	0.069	0.034	0.036	0.008	0.000	0.000
Lower bound	−0.132	−0.065	−0.071	−2.057	−0.999	−1.073
Panel B. First-stage estimation				MHI-1	MHI-2	MHI-3
Upper bound				0.472	0.968	0.899
Lower bound				0.128	0.264	0.247

Table A3 Panel A presents the confidence intervals of the estimated impacts of maternal mental health on children’s vocabulary at the age of 5 years obtained from Equation (2), using Ordinary Least Squares (OLS) and Instrumental Variables (IV) as estimation approaches. Panel B also presents confidence intervals obtained from the first stage of the IV estimation.

## Appendix D

**Table A4:** Correlations between Shock in 2002 and Maternal Mental Health Index in 2006/2007.

	(1)	(2)	(3)
	MHI-1	MHI-2	MHI-3
Experienced shock of crop or livestock	0.176	0.354	0.335
Loss in 2002	(0.126)	(0.256)	(0.239)
Observations	1095	1095	1095
Mother controls	Yes	Yes	Yes
Child controls	Yes	Yes	Yes
Household controls	Yes	Yes	Yes
R-squared	0.074	0.075	0.076

Table A4 presents correlations between maternal mental health indexes and the main shock under analysis experienced by mothers of our sample. The sample is restricted to our group of interest. Mental health indexes are the standardized measures described in the data section using the SRQ-20 items.
